# Supplementary material for: Incidence of extraovarian clear cell cancers in women with surgically diagnosed endometriosis: A cohort study
Source: PLoS One. 2021 Jun 29;16(6):e0253270. doi: 10.1371/journal.pone.0253270 (PMC8241117; doi:10.1371/journal.pone.0253270)
Supplement: S1 Table — (DOCX) [file pone.0253270.s001.docx]

**S1 Table. Type of endometriosis according to the International Statistical Classification of Diseases and Related Health Problems versions 9 (ICD-9) and 10 (ICD-10).**

| **TYPE OF ENDOMETRIOSIS** | | **ICD-9** | **ICD-10** |
| --- | --- | --- | --- |
| **Ovarian**^a)^ |  | 6171A | N80.1 |
| **Peritoneal**^b)^ | Tubal | 6172A | N80.2 |
|  | Peritoneal | 6173A | N80.3 |
|  | Retrouterinal | 6173B | - |
| **Other^c)^** | Rectovaginal | 6174A | N80.4 |
|  | Intestine | 6175A | N80.5 |
|  | Bladder | - | N80.80 |
|  | Sacrouterine ligaments | - | N80.81 |
|  | Cicatrix cutis | 6176A | N80.6 |
|  | Other specified | 6178X | N80.8, N80.89 |
|  | Other unspecified | 6179X | N80.9 |

^a)^ Possible additional diagnosis: ICD-9: 6172A, 6173A, 6173B, 6176A, 6178X, 6179X; ICD-10: N80.2, N80.3, N80.6, N80.8, N80.89, N80.9

^b)^ Possible additional diagnosis: ICD-9: 6176A, 6178X, 6179X; ICD-10: N80.6, N80.8, N80.89, N80.9.

^c)^Including ovarian endometriosis concomitant with endometriosis of rectovaginal, intestine, bladder or sacrouterine ligaments.
