# Supplementary material for: Incidence of extraovarian clear cell cancers in women with surgically diagnosed endometriosis: A cohort study
Source: PLoS One. 2021 Jun 29;16(6):e0253270. doi: 10.1371/journal.pone.0253270 (PMC8241117; doi:10.1371/journal.pone.0253270)
Supplement: Attachment — Submitted filename: RESPONSE TO REVIEWERS290421LS.docx [file pone.0253270.s002.docx]

**RESPONSE TO REVIEWERS’ COMMENTS**

Thank you for your comments concerning our manuscript.

Please find enclosed our replies and revisions written using red font.

5. Review Comments to the Author

Reviewer #1: This research article is very interesting and answers one important question in endometriosis and oncology.
The document is well written with a clear aim and methodology.
The research includes a large group population and this obviously contributes on the strength of the research
All the current researches on this topic have been correctly cited in the article and discussed on the discussion session.

Thank you for these kind comments.

Reviewer #2: The present study aims at evaluating the association between extraovarian clear cell carcinomas and the diagnosis of endometriosis. I have several concerns regarding the consistency of this study and in my opinion the results and conclusions do not reliably represent an original finding on this topic.
- Some of the limitations of the present study are typical of all studies derived from population based national registries

o the diagnosis/coding might not be punctual and patient

All health care providers are obliged to provide the information to nationwide registries on hospital admissions and cancer diagnoses. These registries have been validated and are known to be reliable. The quality of the Nordic Cancer Registries has been described in Acta Oncologica, Nordic Cancer Registries – an overview of their producers and data comparability, by Pukkala et al. 2018.

In addition, validity of the endometriosis diagnosis in the Finnish Hospital Discharge Register has been proven highly reliable (97% and 95% in bidirectional quality assessment (Trends in the incidence, rate, type and treatment of surgically verified endometriosis – a nationwide cohort study by Saavalainen et al. 2018]).

The registry-based study approach does not only have limitations but also has obvious strengths as compared, e.g., to questionnaire-based data collection. The registries used in our study cover unselected population of whole Finland (no selection bias), and the follow-up for emigration and death is complete (no lost to follow-up cases). Data linkages are done by using the personal identity codes, which are available for all Finnish citizens and permanent residents since the 1960s and used in all registries, and therefore guarantee accurate record linkage (Biobanks and registries in epidemiological research on cancer. Pukkala, E in Dilner J (ed.): Methods in Biobanking. Methods in Molecular Biology, Volume 675. Totowa, Humana Press 2011, pp.127-164).

This was clarified in the methods (page 5, lines 103-105; page 6, lines 118-120; page 12, lines 217-221)

*The cancer data are collected by health care professionals, pathology laboratories, hospitals and institutions providing cancer treatment from all over the country.*

*All linkages were done by using women’s unique personal identity codes, which are available for all Finnish citizens and permanent residents since the 1960s.*

*The Finnish Hospital Discharge Register and Finnish Cancer Registry are known for their completeness and high quality[16, 35]. In a bidirectional quality assessment of the study cohort 179 of 184 cases (97%) the endometriosis diagnosis verified from the patient files had been correctly reported to the Finnish Hospital Discharge Register. In addition, among the 168 patients who were operated for deep infiltrating endometriosis, 159 (95%) were found among the cohort identified from the Finnish Hospital Discharge Register [15].*

o most commonly only the main diagnosis is indicated (as stated by the Authors themselves)

Unfortunately, we do not understand this comment. The cohort of surgically diagnosed endometriosis consists of women having received endometriosis diagnosis either as the main or concomitant diagnosis in a relevant surgical procedure. The cancer diagnoses have all been histologically verified.

o Histopathological review in all these cases is not available. This procedure in case of clear cell histotype, as for other rare tumors, should be mandatory in order to provide a reliable analysis.

The histopathological classification of the clear cell cancers are made by pathologists in Finland. There is close collaboration between the specialists, which increases the accuracy of histopathological typing.

We have stated this previously in page 5, lines 105-107 as follows:

*Data on the* ***histologically verified extraovarian clear cell carcinomas*** *in the cohort of surgically diagnosed endometriosis were assessed by linkage to the Finnish Cancer Registry using the personal identity codes.*

- Another concern is that the reported cases of clear cell tumors might not be really associated with endometriosis (the definition of endometriosis associated tumors in not simply the coexistence of these two conditions but has to fulfill precise histopathological criteria).

We agree. There is no way to conclude whether the clear cell cancers are **causally** associated with endometriosis, or whether they just coexist in the same person. Therefore, our aim is not to use expressions, which would suggest a causality.

The expression we find to be misleading has been corrected as follows (page 10, lines 172-173 and page 11, lines 212-213):

*We found extraovarian cancers of clear cell histology to be very rare after surgically diagnosed endometriosis in Finland,…*

*There are case reports of women with endometriosis who concurrently or later have been diagnosed with clear cell carcinoma in locations such as in vagina, rectovaginal septum, peritoneum, diaphragm, and scars such as abdominal wall, umbilical node, caesarean section, or in episiotomy, or in unknown primary sites.*

Since our study specifically assessed extraovarian clear cell cancer cases after **surgically diagnosed endometriosis**, we now highlight more clearly that the study results are limited to the women with history of surgically verified endometriosis (page 10, lines 172-174 and page 12, lines 224-225):

*We found extraovarian cancers of clear cell histology to be very rare after surgically diagnosed endometriosis in Finland, only 23 extraovarian cancers were identified in the follow-up of 370,000 person-years.*

*Our results can be generalized only to women with surgically diagnosed endometriosis.*

I do not understand the relevance and usefulness of Table 1 for data and results interpretation. To me data analysis , result interpretation , as present in this paper, is not very easy for the reader.

Table 1 aims to clarify the diversity of the diagnostic codes concerning endometriosis. We have changed Table 1 to be included as supplementary material (S1 Table).

We also rewrote part of Result section for clarification (page 8, lines 156-159):

*Altogether 23 extraovarian clear cancers were observed in women with history of surgically diagnosed endometriosis; the expected number of cases based on the incidence rate in comparable women of the Finnish female population was 26 (Table 2). The incidence of extraovarian clear cell cancer in women with endometriosis was not increased in any age category, specific type of endometriosis, or specific site of cancer. All cancer diagnoses had been made more than 5 years after the date of endometriosis surgery.*

The interpretation was renewed in the Discussion (page 10 and 11, lines 172-190):

*We found extraovarian cancers of clear cell histology to be very rare in women with surgically diagnosed endometriosis in Finland, only 23 extraovarian cancers were identified in the follow-up of 370,000 person-years. The two previous studies focusing on clear cell cancers in extraovarian sites with concurrent diagnosis of endometriosis of the same location have been based on pathological review of cases in single institutions [17, 18]. In these studies clear cell and endometrioid carcinomas as well as adenosarcoma have been the most common types of cancer diagnosed concurrently with extraovarian endometriosis. More recently, a survey-based cohort study from Japan – where the proportion of clear cell ovarian cancer is exceptionally high – found one case of clear cell cancer among 1397 women with concurrent endometriosis outside the ovaries and the pelvic peritoneum.[19]. Our study is the first to evaluate the standardized incidence ratios of extraovarian clear cell cancer using population-based registries after surgically diagnosed endometriosis. Hence direct comparison of our findings with the earlier studies is not possible.*

We also removed one sentence from the end (page 13, lines 240-241):

*~~Thus, we conclude that a previous operative diagnosis of endometriosis is not a risk factor for subsequent extraovarian clear cell cancer.~~*

Reviewer #3: I read manuscript entilted "Incidence of extraovarian clear cell cancers in women with surgically diagnosed endometriosis: a cohort study" with great interest. Endometriosis is known risk factor for ovarian clear cell cancer but there is no easy pathological explanation for possible influence of endometriosis on extraovarian clear cell cancer and why it even should be. And it was also proven by the Authors. But this main conclusion should be also supported by data from "general population".
Please describe general population - how it was choosen? what was age structure of general population? what was the incidence of extraovarian clear cell cancer in general population?
I found only statement that "The risk of extraovarian clear cell cancers in women with surgically diagnosed endometriosis is similar to that in the general population." But I did not find any data from general population or clarifications in discussion which can prove this thesis.

We thank the reviewer for these kind comments.

We have clarified the issue of the reference rates used in the calculation of expected numbers of cases as follows: The expected rate for each five-year age category and five-year calendar period stratum is calculated by multiplying the number of person-years developed by the women with endometriosis into a given five-year age category and five-year calendar period by the incidence rate of extraovarian carcinoma in the respective five-year age category and five-year calendar period in the entire Finnish female population (Methods: page 6 lines 126-130):

*The expected number was defined by multiplying the accumulated person-years of follow-up in each stratum by the corresponding cancer incidence rate in the entire Finnish female population. These reference rates for each five-year age category and five-year calendar period were calculated using data from the Finnish Cancer Registry.*

The other main point to correct is that this study refers only to Finnish population. Please state this clearly in abstract and in the main text. The results of similar study conducted in Asiatic populations may have different results, because of the known higher incidence of clear cell cancer among Asians.

We apologize if the text was not clear with this. The standardized incidence ratio in this study is the relative risk between the incidence in Finnish women with endometriosis and the incidence of same-aged women in the entire Finnish female population. The absolute incidence of extraovarian clear cell carcinoma in the study region should not interfere the relative risk estimate but it would affect, e.g., the estimate of absolute excess risk (AER). AERs were not calculated in our study but it would be important to calculate them in a study comparing excess risks with areas of very different baseline incidence levels.

We have clarified this issue by adding words *in Finland* (Abstract, page 3, line 60; Introduction, page 4, line 83; Conclusion, page 13, line 253)

and emphasizing this in the beginning of Discussion with line (page 10, lines 167-169):

*The overall incidence of extraovarian clear cell cancer in Finnish women with surgically diagnosed endometriosis did not differ from the incidence of same-aged women in the entire female population in Finland, nor did the incidence of clear cell cancer in any specific site of cancer.*

The explanation in discussion section about Finnish Hospital Discharge Register and Finnish Cancer Registry I found very important and supportive.

6. PLOS authors have the option to publish the peer review history of their article ([what does this mean?](https://journals.plos.org/plosone/s/editorial-and-peer-review-process#loc-peer-review-history)). If published, this will include your full peer review and any attached files.


If you choose “no”, your identity will remain anonymous but your review may still be made public.


**Do you want your identity to be public for this peer review?** For information about this choice, including consent withdrawal, please see our [Privacy Policy](https://www.plos.org/privacy-policy).

Reviewer #1: No

Reviewer #2: No

Reviewer #3: **Yes:** Maria Szubert, MD

[NOTE: If reviewer comments were submitted as an attachment file, they will be attached to this email and accessible via the submission site. Please log into your account, locate the manuscript record, and check for the action link "View Attachments". If this link does not appear, there are no attachment files.]

While revising your submission, please upload your figure files to the Preflight Analysis and Conversion Engine (PACE) digital diagnostic tool, <https://pacev2.apexcovantage.com/>. PACE helps ensure that figures meet PLOS requirements. To use PACE, you must first register as a user. Registration is free. Then, login and navigate to the UPLOAD tab, where you will find detailed instructions on how to use the tool. If you encounter any issues or have any questions when using PACE, please email PLOS at [figures@plos.org](mailto:figures@plos.org). Please note that Supporting Information files do not need this step.

*In compliance with data protection regulations, you may request that we remove your personal registration details at any time.* [*(Remove my information/details)*](https://www.editorialmanager.com/pone/login.asp?a=r)*. Please contact the publication office if you have any questions.*
