# Supplementary material for: Incidence of extraovarian clear cell cancers in women with surgically diagnosed endometriosis: A cohort study
Source: PLoS One. 2021 Jun 29;16(6):e0253270. doi: 10.1371/journal.pone.0253270 (PMC8241117; doi:10.1371/journal.pone.0253270)
Supplement: Attachment — Submitted filename: RESPONSE TO REVIEWERS250521.docx [file pone.0253270.s003.docx]

**RESPONSE TO REVIEWERS’ COMMENTS**

Thank you for your comments concerning our manuscript.

Please find enclosed our replies and revisions written using red font.

ACADEMIC EDITOR: The manuscript has been improved. There is a minor issue that should be addressed.

The Authors extensively discussed the types of cancer detected in endometriosis patients in the Discussion but, apart from the table, the Results section does not mention this topic at all. A discussion should be referred to the findings observed. A brief description of the cancer types observed would be useful also in the Results section.

Thank you. Results section very briefly summarizes all our results shown in Table 2.

To highlight that we also studied the specific sites of clear cell cancer added some words in the text. (page 8, lines 156-157)

*Altogether 23 extraovarian clear cancers were observed in women with history of surgically diagnosed endometriosis; the expected number of cases based on the incidence rate in comparable women of the Finnish female population was 26 ~~(Table 2)~~. The incidence of extraovarian clear cell cancer in women with endometriosis was not increased in any age category, specific type of endometriosis, or specific site of cancer* ***(intestine, cervix, uterus, kidney, urinary organs)****. All cancer diagnoses had been made more than 5 years after the date of endometriosis surgery.* ***(Table 2)***

In Discussion the first section presented other similar-like studies, the second association with clear cell cancers in kidney, uterine and cervix cancer, and the third bladder, urethra and intestinal cancers.

We would like to keep that in our manuscript.

Abstract: there is a repetition in the Results

We corrected this as follows (page 3, line 55):

*The endometriosis cohort consisted of 48,996 women, including 22,745 women with ovarian and 19,809 women with peritoneal endometriosis. Altogether 23 extraovarian clear cell cancers were observed during 367,386 person-years of follow-up. The risk of extraovarian clear cell cancer was not increased among all women with surgically diagnosed endometriosis (standardized incidence ratio 0.89, 95% confidence interval 0.56-1.33) nor in different types of endometriosis ~~of endometriosis~~. The incidence of clear* cell cancer in any specific site was not increased either.
